# Understanding the Impact of COVID-19 on People with Severe and Persistent Mental Illness within Rehabilitation Services: A Thematic Analysis

**DOI:** 10.1007/s40737-022-00320-5

**Published:** 2022-11-23

**Authors:** Luke Pervan, Stephen Parker, Maddison Wheeler, Frances Dark

**Affiliations:** 1 Metro South Addiction and Mental Health Service, Brisbane, QLD Australia; 2 School of Medicine, University of Queensland, Brisbane, QLD Australia

**Keywords:** COVID 19, Serious and persisting mental illness, Rehabilitation, Impact

## Abstract

The COVID 19 pandemic has required major changes in healthcare delivery. This study sought to understand the impact of the mental healthcare changes associated with COVID-19 on people living with severe and persisting mental illness (SPMI) and staff working in rehabilitation teams in Queensland in Australia. Telephone interviews were completed with participants diagnosed with SPMI who were supported by the rehabilitation teams of a public mental health service (n = 18). Additionally, an anonymous survey was completed with staff from these teams (n = 20, 17.5% of staff). Both datasets were analysed separately using thematic analysis. Four themes were identified through the analysis of the patient interviews: wishing the whole thing would go away; [COVID-19 has] delayed my recovery; being more socially aware; and (you've) got to be clean (which is a) good thing. Four themes emerged through the analysis of the staff survey data: needing to change the model of care; impact on patients, the impact on staff, positive impact. The perceived impact on participant’s mental health was like that which was reported in the general population. Participants’ emphasised anxiety, loneliness, boredom, and depression rather than a relapse of their primary psychotic illness. Participants noted the pandemic slowed the pace of personal recovery and limited the delivery of specialised rehabilitation programs.

**Supplementary Information:**

The online version contains supplementary material available at 10.1007/s40737-022-00320-5.

## Background

The COVID-19 pandemic is associated with significant morbidity and mortality (Cucinotta and Vanelli 2020), and the public health response has dramatically impacted people’s lives (Fisher et al. 2021). In Australia, physical distancing requirements and restrictions on movement and activity ('lockdowns') have limited community transmission but have detrimentally impacted individual well-being (Fisher et al. 2021). The need to address the mental health impact of the pandemic was recognised early through the National Mental Health and Wellbeing Pandemic Response Plan (National Mental Health 2020). There are special needs groups within the population whom the COVID-19 response may particularly impact as they are already vulnerable and socially marginalised (Eshareturi et al. 2021). One such group is people with severe and persisting mental illness (SPMI) who will predominantly be diagnosed with schizophrenia and related psychotic disorders and have high levels of ongoing associated disability.

### Impact of the Public Health Response on Mental Health and Well-Being

COVID-19 was designated a pandemic by the World Health Organization in March 2020 (Cucinotta & Vanelli 2020). A growing body of research has explored the impact of COVID-19 on the general population's mental health in multiple locations around the world. While there has been considerable heterogeneity between studies (Xiong et al. 2020), a consistent finding has been the high rates of psychological distress, including clinically significant symptoms of anxiety and depression (Batterham et al. 2021; Dawel et al. 2020; J. R. Fisher et al. 2020; Salari et al. 2020; Xiong et al. 2020). In these studies, the presence of pre-existing or chronic mental illness has been identified as a risk factor for higher levels of psychological distress in the context of the pandemic (Batterham et al. 2021; Xiong et al. 2020).

In Australia, surveys undertaken in the early months of the pandemic suggested a higher-than-expected levels of anxiety, depression, and psychological distress (Batterham et al. 2021; Dawel et al. 2020; Fisher et al. 2020). Furthermore, a longitudinal cohort study of people who had a pre-existing mental illness, found that the first 3-months of the pandemic was associated with higher levels anxiety and depressive symptoms (Batterham et al. 2021). In these studies, numerical rating scales were used to quantify data and there remains little published qualitative information about the personal impact on individuals. However, in one large scale survey involving thousands of respondents, it was identified that a personal history of a negative impact of COVID-19 (e.g. job loss) was associated with higher levels of psychological distress (Fisher et al. 2020).

Very little is known about how well public health messages relating to COVID-19 have been received and acted on by people affected by SPMI. A cross-sectional survey of 132 patients with SMPI in India explored impact and understanding of COVID-19 and found most participants had inadequate understanding of symptoms of COVID-19 and precautionary measures. This compared unfavorably to the high levels of public awareness observed in other surveys of the general Indian population (Muruganandam et al. 2020). While it is difficult to generalize from a single study conducted outside of the Australian context, these findings suggest that people with SPMI may struggle to receive and understand public health messaging. Possible reasons for this include clinical symptoms, social isolation, and limited access to technology (Muruganandam et al. 2020).

### The National and State COVID-19 Response in Australia

The Australian health system involves three layers of government. The Commonwealth government is responsible for the universal medical and pharmaceutical benefits schemes. The State and Territory governments are primarily responsible for public health services including public hospitals. Local governments provide environmental health and healthy lifestyle programs. In response to the pandemic, the National Cabinet of Australia was established to ensure a population response to COVID-19 (Department of the Prime & Cabinet, 2021). This brought together the Prime minister, and the State Premiers together for weekly online meetings to help negotiate and communicate the state and the national response.

COVID-19 has spread unevenly in Australia, leading to different policies and public health directives depending on the risk of COVID-19 in a particular state population. Borders between states have been closed in successive lockdowns. Australia entered a “nationwide shutdown” with forced closure of non-essential businesses on 23 March 2020. The State in which the study was undertaken, Queensland had been subject to restrictions on the greater population which included a “lockdown” from March to April 2020, with people only allowed to leave their place of residence for essential reasons (Lynch 2021). After the stay-at-home orders were ended, ongoing restrictions remained in place, including limits on public gatherings, capacity limits for indoor venues, caps on visitors to private residences, and visitors to hospitals and aged care facilities not being permitted (Lynch 2021). The state’s borders remained closed to other regions of Australia where community transmission of coronavirus had been more prevalent. Restrictions were gradually lifted in June and July 2020 (Lynch 2021). However, limits on public gatherings and visitors to hospitals and aged care facilities were reimposed in the Brisbane region in August 2020 due to a relatively small recurrence of community transmission of coronavirus (Lynch 2021). Masks were not mandated for the public in Queensland throughout 2020 except in hospitals during the outbreak of cases recorded in August.

### Changes to Healthcare

The public health response to COVID-19 has altered healthcare delivery in Australia. Videoconferencing for consultations became essential for providing care while maintaining social distancing, especially for vulnerable patients (Health 2020). The aim was twofold: increasing capacity for emergency care and limiting non-essential physical contact that could contribute to the community spread of the virus.

Video or teleconferencing became the primary mode of conducting mental health appointments. (Theodoros et al. 2020). In addition, COVID-19 screening processes were used before face-to-face assessments in inpatient and community mental health facilities. Personal protective equipment (PPE) was made available to staff and consumers. Additionally, measures were taken at the individual mental health service level to address the emerging risks. For example community staff were divided into sub-teams that alternated working from home or at the clinic to minimise transmission within the teams (Theodoros et al. 2020). Audits were conducted to check what electronic devices patients had that could deliver non-face to face care. The staff made plans with patients to ensure they understood the change in care delivery, agreed to ongoing contact with the service and would manage during social isolation.

Only a small proportion of people with severe mental illness require the intensive support of a specialized mental health rehabilitation team (Meehan, Stedman, Parker, Curtis, & Jones, 2017). This patient group is generally characterized by high levels of persisting symptomatic and functional impairment that limits their independence in the community. Mental health rehabilitation services generally focus on patient's skill development and evidence-based therapeutic programs conducted individually or in a group setting. The COVID-19 response resulted in the cancellation of group therapeutic programs (Dark et al. 2021). Additionally, the shift away from face-to-face support impacted the direct observation, modelling, and coaching involved in living skills development.

### Objectives

This study sought to understand the impact of mental healthcare changes associated with COVID-19 from the perspectives of patients with SPMI in the rehabilitation teams of a public mental health service and the staff employed in these services. This study also explored whether people with SPMI engaged with rehabilitation teams understand and act on the public health messaging related to COVID-19.

### Research Questions: Patients in Rehabilitation Teams

The research questions were written for this study by the authors to ascertain what patients knew about COVID-19; their source of information; their understanding of the behavioural change required to minimising acquiring and transmitting COVID-19; their views on the changes made to healthcare delivery in response to COVID-19 and the impact of these changes on their mental health and wellbeing.

1. What do patients in rehabilitation teams know about COVID-19?
2. How have patients acquired their knowledge?
3. Do patients know what social distancing means and have they been able to social distance?
4. What is the patient perspective on the change in mode of healthcare delivery due to COVID-19?
5. What is the patient perspective of the impact of COVID-19 and the changes to healthcare delivery on their mental health and wellbeing?

### Research Questions: Staff Working in Rehabilitation Teams

The research questions were written for this study by the authors to ascertain the impact of COVID-19 on the capacity of staff in the rehabilitation teams to deliver care as well as their views of the impact on patients.

1. What changes have occurred in your work life due to COVID-19?
2. What impact has COVID-19 had on your ability to deliver care?
3. What is the staff perception of the impact on patients of changes resulting from COVID-19?

## Methods

This qualitative study explored multiple stakeholder perspectives (patient and staff) of the impact of COVID-19 on mental health rehabilitation services. All staff working in the rehabilitation teams were made aware of the study via email with a link to the online survey. Patient participants were informed of the study via their treating clinicians in the community and via weekly community meetings in the residential sites. Semi-structured interviews with mental health rehabilitation patients were conducted by 2 of the authors (FD, LP) via teleconferencing (09–12/2020). The interviews were conducted by phone due to COVID19 restrictions and were completed within 30 min. Additionally, staff perspectives were obtained using an anonymous online survey over the same time. Finally, a thematic analysis of stakeholder data was completed. The study and its findings were reported considering the COREQ checklist (Booth, Hannes, Harden, Noyes, & Harris, 2014) (see Supplementary Materials). All participation was based on voluntary informed consent, and ethics approval was obtained before study commencement (HREC/2020/QMS/64496).

### Participants

A minimum target of 15 in-depth patient interviews was set; a number exceeding the threshold of 12 interviews that is generally adequate to achieve higher levels of thematic saturation (Guest et al. 2020). All participants needed to be competent in English at a level of 4 years of education. The researchers took notes of the interviews which were not recorded. The notes were read back to the participant for verification that they represented the participant’s views.

In addition, all staff employed in the study rehabilitation services in September 2020 were emailed to be informed of the study by their team leader and invited to complete the anonymous online survey.

### Analysis

De-identified transcripts were uploaded to NVivo12 for analysis. The theoretically flexible approach of Braun and Clarke (Braun & Clarke 2006) was used in this analysis. Braun and Clarke describe two main approaches; a top-down theoretical thematic analysis driven by specific research questions and a bottom-up inductive analysis that is driven by the data. In this study the research questions framed the data collection, but the analysis of the data followed a bottom-up inductive approach. These steps are not linear but part of a recursive analysis of the data. There were two main data coders who familiarised themselves with the data before coding each item and collating the codes and data extracts. Each coder searched for themes which were discussed with a third author. Once consensus was gained on the themes the coded data was sorted. The themes were reviewed with reflection on the “story” told by this analysis. The emergent themes were defined and named based on how they represented the data and contributed to the overall “story”. The emergent content and themes were discussed with three of the authors with excerpts chosen by consensus of all the authors.

## Results

All patients of the rehabilitation services were made aware of the study by their treating team with 18 people agreeing to participate and being interviewed. The response rate was 25% (n = 5) for Bayside Residential Rehabilitation unit (RRU), 31% (n = 5) for the Logan RRU, 20% (n = 4) for Coorparoo RRU and 5% (n = 4) for the Mobile Intensive Rehabilitation Team (MIRT) (Table 1). The 18 participating patients included 9 male and 9 female patients. The median age of participating patients was 41 years. Detailed patient demographic data was not collected for this study. The average length of stay in a RRU is 12 months. The sample was skewed with most respondents, (77.7%) from the three RRUs. This study sample was older (median age 41) than the average age for RRU (31.4 years) (Parker et al. 2019) and MIRT (32.5 years) (Siskind et al. 2021).

**Table 1:** Mental Health Rehabilitation team descriptions, staffing numbers and response rate of consumers per service

Site	Staff employed n =	Service description	Patient Population in teams	Patient participating in the interviews	Response rate
Bayside *RRU	24	Community-based recovery-oriented residential mental health rehabilitation support focusing on living skills development and community integration	20	5	25%
Logan RRU	21	Community-based recovery-oriented residential mental health rehabilitation support focusing on living skills development and community integration	16	5	31%
Coorparoo RRU	16	Community-based recovery-oriented residential mental health rehabilitation support focusing on living skills development and community integration	20	4	20%
**MIRT Logan	13	Community based intensive mental health rehabilitation support based on an adaptation of the Assertive Community Treatment model	45	0	
MIRT PAH	14	Community based intensive mental health rehabilitation support based on an adaptation of the Assertive Community Treatment model	80	4	5%
***THT	9	Provides transitional accommodation in community based independent living units, and intensive domestic and community support to consumers for up to a six-month period	16	0	
Wisteria	17	Sub-acute medium term inpatient mental health extended treatment and rehabilitation unit for adults aged 18–65	16	0	

The staff survey was anonymous, and their work site not indicated

*Residential Rehabilitation Units

**Mobile Intensive Rehabilitation Teams

***Transitional Housing Team

Twenty of the 114 staff (17.5%) responded to the anonymous survey. No demographic or work location details were collected on staff to ensure anonymity.

### Qualitative Analysis of Patient Interviewees

Four main themes emerged from the qualitative analysis of the patient interviews.

#### Theme 1. "I Wish the Whole Thing Would Go Away"

The participants accepted the immediate situation and the need for precautions such as social distancing. There also seemed to be an awareness that restrictions were necessary to protect the community as well as to keep personally safe. There was a hope that the situation would be transient and soon come to an end.

“[My goal] is to stay away from COVID-19 so I can still see my children…Pandemic so doing what I needed to do” (Participant 5).

"Outings couldn't go ahead, and visitors stopped. I get lonely and wish the whole thing would go away" (Participant 1).

#### Theme 2. "COVID-19 Restrictions Delayed my Recovery"

There was recognition that the service was doing their best to adapt care delivery while incorporating COVID-19 prevention strategies but also that the comprehensive recovery-based rehabilitation program was compromised. The participants made the distinction between clinical care and aspects of the rehabilitation program that was part of their recovery. The restrictions impacted particularly on the recovery group programs.

“It [COVID 19 restrictions] has slowed my recovery because I have decreased contact with people.” (Participant 3).

“It restricted a lot of things. Cancelled Cogsmart [Cognitive Compensatory Program], cooking groups, various classes couldn’t run during lockdown. Very little happening …disappointing…… [It has] delayed my recovery. I was going to the gym twice a week but can’t be due to the lock down. It [the lock down] held me back from finding hobbies, job friendships and stuff. Recovery does not complete without hobbies. Pretty ok but [COVID -19 lockdown] has stopped things from progressing." (Participant 6).

“More social aspects of RRU stopped or change for example art therapy, exercise, cooking groups…we were encouraged to stay in our units. They did check on us in terms of our mental health, but I got “cabin fever…. Slowed my recovery but they tried their hardest.” (Participant 7).

“Inability to move forward with my care plan for example exposure therapy.” (Participant 8).

#### Theme 3. More Socially Aware

The response to the pandemic has required a population response that has highlighted issues such as a sense of citizenship and ethical issues of the rights of the individual versus the rights of the community. The respondents commented on concerns they had not just for themselves but for their family and vulnerable members of the society.

"I am more socially aware. I took certain freedoms for granted before, now I take advantage that I take control of 95% of what I do" (Participant 7).

“I am worried about [my] family in Victoria…Grandparents hardly able to leave the house. Great grandparents in New South Wales worried I can’t see them”. (Participant 6).

"Most people who get it come through, but a percentage don't especially people who are immune compromised". (Participant 6).

“[I am] trying to support businesses in Queensland …I worked in hospitality in the 90’s…I feel for people who have lost their jobs”. (Participant 5).

“[I am] worried about [my] father who has had a fall and two strokes… I want to support mum. (Participant 3).

#### Theme 4 "[You have] Got to be Clean… [Which is a] Good Thing"

In addition to the responsibility to others noted in Theme 3, respondents were aware of taking personal responsibility for their behaviour. Generally, the respondents understood the rationale for the health precautions and even acknowledged some positive change.

“Staff communication about COVID was good. Some slight anxiety when people got (COVID) tested…(but) scared when staff wore gowns and masks" (Participant 1.)

"I am…. more mindful where I am what I'm doing or what I am touching. sanitisation is so huge. [There are] good aspects… not taking it for granted." (Participant 7).

“I got a clear insight about what to do concerning safety precautions …just got to be aware where I am and what I am doing. Tended to forget… signs and displays helped.” (Participant 9).

### Other Observations from Qualitative Patient Data

Most patients (15, 83%) described that COVID-19 is a contagious virus during their interviews. Many (8, 44%) acknowledged that COVID-19 primarily causes respiratory symptoms, can be deadly, and that elderly and immunocompromised persons most at risk. All the patients demonstrated awareness of COVID-19 and very few reported incorrect information. Most patients (16, 89%) reported receiving information from mainstream news sources. Many reported receiving information from friends and family (9, 50%) or their clinical care workers (9, 50%).

All patients demonstrated an understanding of social distancing and indicated that they were trying to adhere to this. Most (15, 83%) quoted 1.5 m as the social distance between persons (which was consistent with government guidelines at the time).

Most patients (14, 78%) reported either no change to face-to-face appointments or slightly reduced. Few commented on the use of Telehealth, though it should be noted that 14 participants were recruited from residential settings where use of Telehealth was limited and staff continued to attend the premises during periods of lockdown, as they were considered essential workers.

### Qualitative Analysis of Staff Surveys

Twenty staff responded to the survey, and five main themes emerged.

#### Theme 1. The Need to Change the Model of Care

The loss of group-based interventions had a significant impact on the recovery-based specialist rehabilitation program offered.

"It has been difficult having to reduce face to face contact with consumers…I feel this has negatively impacted the ability to work intensively on rehabilitation goals”. (Staff Respondent 1).

“…less opportunity for in vivo learning of ADL’s (activities of daily living), restrictions on group activities, now Queensland Health budget is drying up with knock on effect likely in rehab staffing” (Staff respondent 4).

“…lower standard of care. This is more apparent in community rehabilitation where group activities have been cancelled until further notice”. (Staff Respondent 5).

#### Theme 2. Impact on Patients

Staff commented that the impact of the changes appeared to differ based on the individual patients, their care setting (residential versus community), and the intervention (group versus individual).

“It depends on the patient. In the community rehabilitation setting the progress of many patients has been negatively affected by COVD-19. In these cases, the restrictions on gatherings have had a greater impact than fear of the virus itself. Group activities which many patients found to be an important part of their rehab activities had to be cancelled. Some patients lost momentum that they had been building in rehab and their newfound boredom seemed to have led them to resume old unhelpful habits e.g., gambling, smoking and drinking (in a few cases).” (Staff Respondent 7).

“(The impact of COVID-19 has been) negative with increased anxiety, social isolation and less opportunity for social and leisure activities outside of the home.” (Staff respondent 4).

Staff responding appeared attuned to the impact on consumers and how this changed overtime. The positive behaviour change was also noted by staff.

"There was initially a lot of anxiety about COVID and concerns about family by the residents, which settled overtime and with support. The other major negative impact was around not having visitors nor being able to leave to visit family for a period. Caused some distress to residents and families, but all understood. There has been positive awareness of hand hygiene etc. so less general illness in residents. (Participant 2).

#### Theme 3. Impact on Staff

Staff commented on their well-being related to work changes and the impact on their life outside of work, especially routine leisure activities.

"[There has been] limited change. Self and team are still at work, not working from home as it is a residential setting. Negative impact of more meetings, guidelines, planning and more sick leave of staff needing to get tested if they have cold." (Staff Respondent 2).

"Overall [my work life] has changed negatively. In April and May my workload have increased due to staff required to take unexpected leave for COVID-19 testing… Work life has been disrupted as I am unable to conduct many leisure activities outside of work… I have experienced considerable mental fatigue from the near constant news and updates regarding coronavirus, wildly incorrect predictions, uncertainty about the future, separation from friends and family who are mostly abroad and at times frustration over the inconsistent and haphazard way authorities have responded to this crisis. Overtime my attitude toward coronavirus has shifted increasingly towards indifference and favouring promoting quality of life over prolonged restrictive measures." (Participant 5).

#### Theme 4. Positive Impact

Prior to the pandemic telehealth had not been part of routine rehabilitation care delivery. The "pivoting" away from relying on face-to-face contact with consumers was considered a significant change to the ideal care delivery for mental health rehabilitation. Staff were also able to see the positive outcomes of the forced change to using technology.

"The up-take of tele-health—I didn't expect people to be able to adapt so quickly, given everyone is at different stages of tech-literacy. Overall, I think the service has done very well being forced to 'think on our feet' to solve the problems quickly. I also think the ability for meetings to happen via Teams because of the restrictions has been positive and has made information more accessible. (Staff respondent 1).

I think there will be long lasting positives in terms of clinicians now able to connect with consumers via phone/telehealth and using technology to deliver interventions creatively. However, I do not think that this is a replacement for the regular model of service. (Staff participant 1).

The positives (personally) have been better work-life balance with an ability to work remotely. (Staff Respondent 1).

“Positive [impact on patients] in relation to hand hygiene …can benefit … Personal space is now practiced by using social distancing which is helpful for people who don’t like others in their personal space plus it will mean people need to consider others more than they have previously, which is positive in my view.: (Staff Respondent 3).

…. Positive aspects are limited but there are anecdotal reports of less supply of illicit drugs, with positive impacts on mental health.” (Staff respondent 4).

## Discussion

Responding to the COVID19 pandemic has required public health messaging that permeates to all in the population and is effective in mass behavioural change (Bavel et al. 2020). The pandemic response has meant changes on an individual but also organisational level (Lee et al. 2021). Disparities in health literacy has been considered a factor in the uptake of public health messaging and the behavioural change being communicated (McCaffery et al.). The results from this study suggested good health literacy in the participants regarding COVID-19. There was no inaccurate beliefs and the behavioural responses required regarding social distancing were adhered to. Most respondents were in residential rehabilitation units with frequent staff contacts to assist with consolidation of key messages and clarification of behavioural requirements.

The patient participants were aware of the necessary compromise in the specialist rehabilitation programs but also perceived that their recovery was impeded by these changes. The focus of care in mental health rehabilitation is on improving function. There was a distinction made between clinical care being able to be continued but recovery orientated, and community functioning interventions being restricted. The patient participants missed the group therapies but also social activities that they valued as part of their recovery. This concern is being reflected in the literature on the impact of COVID-19 on psychosocial interventions (Judith A. Cook & Jessica A. Jonikas, 2020). Pivoting care to minimise face to face interventions has highlighted the digital divide (Judith A. Cook & Jessica A. Jonikas, 2020) not only in who has access to the technology but the divide in the type of care that can currently be delivered in this mode. The staff survey responses had very similar themes to the consumer responses. Staff also worried about the impact on specific rehabilitation interventions especially if the restrictions were extended. They were able to see some positive change from the crisis with rapid uptake of the use of technology even among previously resistant staff. Knowledge about the burden on healthcare staff of working during pandemics, including COVID-19 have been the subject of a systematic review (Billings et al. 2021). The specific issues for staff as well as patients in recovery and rehabilitation services is the focus of current research (Rydwik et al. 2021).

The has been a rise in mental well-being awareness in the community due to the ongoing pandemic (Kinman et al. 2020; Rossell et al. 2021a). In an interim report from the COVID-19 and you: mentaL health in AusTralia now survEy (COLLATE), the authors identify a common concern being for the welfare of loved ones, of increased negative emotions of anxiety, stress and depression and being a young female and having an existing mental illness (Rossell et al. 2021a). The early recognition of the impact of the pandemic on mental health because of the pandemic influenced the establishment of the National Mental Health and Wellbeing Pandemic Response Plan. The patient participants concerns were in line with what has been found for the general population with anxiety and a concern for loved ones (Rossell et al. 2021b). The staff talked about the challenges they faced looking after their own health and well-being with restrictions limiting usual leisure activities. The theme from the consumer participants of "(I) Wish the whole thing would go away" picked up on the tone of responses from both groups while coupled with an acceptance of doing what is required to stay COVID safe**.**

### Clinical Implications

In the response to the pandemic provision of accurate information about COVID-19 delivered by trusted sources is essential. Simple messaging that is available in key public areas (e.g., floor markings indicating social distance) assists in facilitating group behavioural change.

Personalised information and support are also required that respects that people with SPMI may also have concerns for their dependents (children and elderly parents) or clinical vulnerabilities (e.g., substance misuse, social anxiety, co-morbid physical illness) that may make them vulnerable to adverse reactions to COVID-19.

### Future Directions

Responding to COVID 19 has highlighted how dependent psychosocial mental health rehabilitation is on face to face and group work. There is a need to develop evidence in how existing therapies addressing functional recovery can be adapted for online use (Dark et al. 2021).

### Limitations

The results from this study cannot be generalised other mental health rehabilitation services nor to the broader population of people with severe mental illness. The sample was skewed towards residential rehabilitation services with a relative underrepresentation of community rehabilitation and people being cared for in the transitional housing team which helps house homeless people with severe mental illness. In addition, the lack of detailed demographic information and small sample size meant that factors such as ethnicity and marital status could not be accounted for.

The research was conducted by staff-participant researchers who were aligned with the study rehabilitation services. This may have influenced recruitment and responses. Some of the teams are small and to ensure anonymity no demographic data was collected on the staff respondents and limited demographic data collected on patient participants.

Participants did not verify the transcription. The interviewers read back the notes of the interview to the patient participants who were not provided with a written copy. This may not have enabled the participants time to consider their responses and make any changes.

## Conclusion

For the consumers of these Australian mental health rehabilitation services, the challenges they have faced in the COVID-19 pandemic mirror those reported for the population generally. However, they also reported specific impacts on their rehabilitation care and recovery journeys. Ongoing attention is needed to ensure that the rehabilitation needs of people affected by SPMI are not neglected in the focus on maintaining the health and wellbeing of the nation. This will require commitment to the existing staffing and funding of these non-acute rehabilitation services despite the fiscal challenges on health services of COVID-19.

## Supplementary Information

Below is the link to the electronic supplementary material.

Supplementary file1 (DOCX 14 kb)

## Data Availability

Any request for access to de-identified transcripts or survey response data would be subject to approval from the relevant ethics committee.
