# Supplementary material for: Understanding the Impact of COVID-19 on People with Severe and Persistent Mental Illness within Rehabilitation Services: A Thematic Analysis
Source: J Psychosoc Rehabil Ment Health. 2022 Nov 23:1–11. Online ahead of print. doi: 10.1007/s40737-022-00320-5 (PMC9686248; doi:10.1007/s40737-022-00320-5)
Supplement: Supplementary file 1 — Supplementary file1 (DOCX 14 kb) [file 40737_2022_320_MOESM1_ESM.docx]

**Supplementary material: Patient (A) and Staff (B) interview questions**

**A: Patient Participant Questions**

1. What do you know about COVID-19?
2. How did you come to know about COVID-19? (e.g. Family, Friends, The news, online)
3. Thinking about what you know about COVID-19, have you heard about the term socially distancing?

If yes: What does the term social distancing mean to you?

Have you engaged in social distancing?

If no, are there reasons why you haven't participated in social distancing?

1. The mental health service has made changes because of COVID-19, including providing fewer face-to-face appointments. What has this meant for you?

Questions to help people who have trouble responding to open ended questions due to their illness

1. Have you still been seeing your case manager or doctor face to face?
2. Has the frequency with which you are seeing your treating team changed?
3. Do you believe you are getting the mental health care that you need?
4. Do you think the changes that have come about because of COVID-19 have impacted on your mental health?

If yes can you say in which way?

**B: Staff Participant Questions**

1. How has your work life changed either positively or negatively as a result of COVID-19? Please give examples.
2. Do you feel you have been able to deliver the care your patients needed? Please elaborate on your response.
3. Do you think the impact of COVID-19 has positively or negatively affected your patients? If so, can you give examples?
4. Do you think the impact of COVID -19 will have long lasting positive or negative changes in how mental health rehabilitation is delivered in the service? Elaborate on your responses.
